# Disease and the Dynamics of Food Webs

**DOI:** 10.1371/journal.pbio.1000209

**Published:** 2009-09-29

**Authors:** Wayne M. Getz

**Affiliations:** 1 Department of Environmental Science, Policy and Management, University of California Berkeley, Berkeley, California, United States of America; 2 Mammal Research Institute, Department of Zoology and Entomology, University of Pretoria, Pretoria, South Africa

## Abstract

What models and statistical tools can best help us assess how ecosystems respond to the impact of multiple factors, such as disease, predation, fire, and rain?

Fifty years ago, ecologists Hairston, Smith, and Slobodkin [1] proposed the provocative idea that herbivores are limited by predators rather than food. If true, the implications are that we live in a green (lots of uneaten plants) rather than a brown (the bare earth is visible due to overgrazing) world [2]—which is largely, though not universally true. This idea has come to be known as the trophic cascade hypothesis (TCH), and is a seminal idea in the subfield of ecology known as foodweb theory [3]. The TCH generated numerous studies on whether such tritrophic, and even longer aquatic [4], food chains are truly controlled from the top down (the TCH) or from the bottom up through food-limiting herbivore populations [2],[5], even if only at critical points in time. The importance of such studies to our understanding of the responses of ecosystems to land use and climate change, as well as to the ecological implications of emerging disease, will become apparent in this primer.

Like all dialectical arguments, the debate on whether food chains are controlled from the “top down” or “bottom up” is only useful for finding the relative influence of both types of control as they may relate to other ecological, environmental, and particularly seasonal factors. The world, of course, is much more complicated in that identifiable food chains are hardly ever sufficiently isolated from other ecological processes (Figure 1) [6] for models of such processes to make reliable long-term predictions. Omnivores [7], for example, distort food chains by feeding at several trophic levels, whereas microbes feed at all levels: at the bottom as detritivores [8], without which all trophic chains would soon run out of essential resources, and at other levels as parasites and pathogens, often constituting a substantial fraction of ecosystem biomass [9].

**Figure 1:**
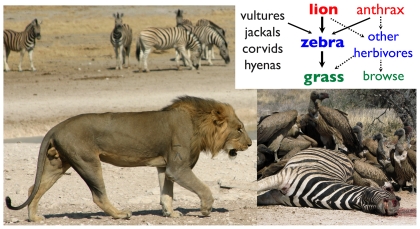
Disease mediation of a lion-zebra-grass tritrophic chain in the Etosha National Park ecosystem in Namibia. The anthrax pathogen, *Bacillus anthracis*, accounts for a substantial number of deaths of zebra and other herbivores (including springbok, elephant, wildebeest), thereby providing a largess of resources for the scavenger community dominated by jackals, hyenas, and several species of vultures and corvids, not to mention lions themselves. (In the lower right picture is a zebra carcass a few hours after death from anthrax with vultures and jackals the first to arrive on the scene). Thus the microbe *B. anthracis* plays an important role in determining the ultimate structure of the Etosha herbivore-carnivore-scavenger foodweb.

## How To Model Trophic Chains

Mathematical models are used to explore questions regarding what factors tip the balance in favor of top-down or bottom-up control. The most versatile models from a trophic point of view are those that take a consumer-resource perspective, irrespective of the particular trophic level under consideration (Box 1): such formulations have utility in modelling how plants extract photons and nutrients from the environment, herbivores consume plants, carnivores consume herbivores, insects consume insects, fish consume fish, and macroparasites (e.g., nematodes, cestodes) extract biomass from most vertebrates. By setting up equations that for each component of a foodweb account for the dominant links among all components, scientists can build models of trophic dynamics that address a plethora of interesting questions. This includes the focal question addressed by Holdo et al. in this issue of *PLoS Biology* [10]: in the competition between trees and grass for space, what is the relative importance of top-down effects exerted by fire and elephants versus bottom-up effects of rainfall and rising CO_2_. Further, and more specifically (as illustrated in Holdo et al.'s Figure 4B), they assess the relative importance of the human-elephant-tree cascade (influenced by poaching) compared with the rinderpest-wildebeest-fire-tree cascade that has resulted from the near eradication of rinderpest—a highly contagious measles-like virus the infects cattle and buffalo, giraffe, kudu, wildebeest, and other artiodactyls—through the vaccination of cattle in east Africa in the early 1960s.

**Box 1. Extraction-growth models**

In the simplest trophic foodweb models, each component is represented by a single variable, be it the biomass density, population density, or some other appropriate measure of abundance or amount. Consider the case where *x_i_* represents some measure of the size of component *i* in a foodweb. Suppose *x_i_*_−1_ is the amount of resources available to individuals in component *i*, and *x_i_*_+1_ is the amount of component *i*+1, containing individuals that consume component *i*. (Note that components may be functional groupings of individuals from several species or even specific phenotypes within same species). If the following three conditions hold [30]–[32]:

1. the amount that each unit in component *i* is able to extract from component *i*−1 is 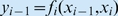

2. the per unit (e.g., per capita) growth rate of the component *i*, which from calculus is represented by 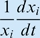 is a function *g_i_* of its per unit consumption rate *y_i_*_−1_ (see [5] for a more detailed elaboration of *g_i_* and [33] for inclusion of a storage component);
3. component *i* is extracted by component *i*+1 at a total (per unit×*n* units) rate is *x_i_*_+1_ *f_i_*_+1_(*x_i_*,*x_i_*_+1_) (from condition 1 above the total amount transferred from *i* to *i*+1 is *x_i_*_+1_ *y_i_*) then the total rate of change of component *i* is governed by the consumer-resource equation (Figure 3)
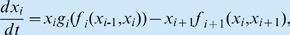
(1)where, the lowest trophic level, *i* = 1, is modelled in terms of the underlying resource being a constant or a time varying input *x*_0_(*t*) (e.g., photon or nutrient flux), or satisfies some type of resource pool dynamics [30]–[32]. First consider the idealized case of a population *x*_1_ = *x* extracting resources from a constant resource flux *x*_0_ = *R*. If:
4. the per unit extraction rate is given by [34],[35] 
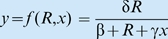
, where δ is the maximum extraction rate and β is the resource amount at which the extraction rate is half its maximum when the intraspecific interference competition parameter *γ* is 0 (where interference competition is an increasing function of the parameter *γ*≥0);
5. the growth rate is the hyperbolic (i.e., metaphysiological) function [30],[31] 
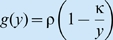
, where ρ is the maximum per unit growth rate when resources are essentially infinite and κ is the metabolic breakeven point (minimum per unit consumption rate needed to avoid decline),
6. the population itself is free from predation, then the governing equation reduces to the ubiquitous logistic growth equation
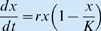
 with 
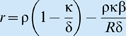
 and 
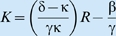

Extending this case to a two-trophic case of an idealized population *x*_2_ exploiting a second idealized population *x*_1_ = *x* that itself is extracting resources from a constant resource flux *x*_0_ = *R*, if both populations grow hyperbolically on resources extracted at rates given by functions conforming to the function given in condition 4 above, then we have the two trophic model:
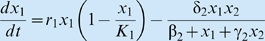
with 
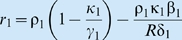
 and 
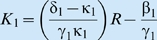


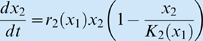
with functions 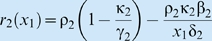 and 
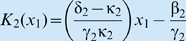
.

This 2-D consumer-resource equation, unlike the usual Lotka-Volterra (L-V) equation, has the internal logic of applying the same growth and extraction principles at both trophic levels. In contrast, the L-V equation arises by assuming that the second trophic level either consumes the first at a rate *f*_2_(*x*_1_,*x*_2_) ≡ *f*_2_(*x*_1_) = δ_2_ *x*_1_ (mass action) or 
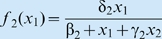
 (modified Holling Type II, see [36]) and that growth is a linear function *g*_2_(*f*_2_) = *cf*_2_−*d* of the feeding (extraction) rate [30]. With this transparency of the assumptions needed to construct an L-V model from Equation (1), we are able to better assess the limitations of L-V models and move to biological more realistic formulations of consumer-resource processes (e.g., see [37]).

## Episodic Versus Steady Processes

Holdo et al. [10] highlight two very different classes of processes that potentially influence the ecological state or regime in which a particular system resides [11]—for example, whether a particular ecosystem functions predominantly as a grassland with isolated trees or as a woodland interspersed with patches of grass, with all the attendant differences in guilds of birds, mammals, and insects that are supported, not to mention plants, fungi, and bacteria.

The first class of processes are episodic perturbations such as outbreaks of fires and diseases that “burn” their way through systems, or environmental switches such as those driven by ENSO (El Niño-Southern Oscillation); although we need to bear in mind that what might be episodic and intense at one spatial scale appears as more regular and less intense at a larger spatial scale. Irregular episodic events (best characterized in terms of probability distributions describing the frequencies and intensities of occurrences) can determine the dominant state of ecosystems even when such events are infrequent [12].

The second class of processes relates to species that extract resources from underlying trophic levels at relatively steady rates (Box 1). If removal of one such species causes a change in the structure of an ecosystem that is highly disproportional to the original biomass contribution of the species to the ecosystem, then the species is known as a keystone species [13]. Some examples are: growing populations of elephants in southern African parks are reducing tree-canopy cover [14] and hence bird diversity [15]; the reintroduction of wolves in Yellowstone National Park is causing elk to avoid predation by remaining at higher elevations, thereby facilitating the recovery of aspen at lower elevations [16]; and the demise of cod in the Baltic sea through overfishing has caused the system to switch to a sprat-dominated regime [17]. Endemic diseases also fall into this latter class, with Holdo et al. [10] demonstrating an ecosystem regime shift in the Serengeti in east Africa. Using discrete time models fitted to population time-series data (Box 2), they show that suppression of endemic rinderpest in the wildebeest population caused the Serengeti ecosystem to increase its woody component by a factor of two to three. Consequently the Serengeti has switched from a net carbon source to a net carbon sink over a 40-y period.

**Box 2. Discrete models and data**

All biological systems constitute a mixture of idealized continuous processes such as expectations of time of death, and idealized discrete processes—typically diurnally or seasonally cued, such as the timing of reproduction in many temperate species. Data, however, are invariably discrete and relate either to counting events occurring within demarcated intervals of time or space, to measuring traits categorized into discrete classes, or to rounding measurements to a given number of significant digits. A statistically oriented approach is to fit the parameters of discrete time equations to sets of time-series data {*x_i_*(1), *x_i_*(2),…, *x_i_*(*T*)}, one set for each component *i*, *i* = 1,…,*n*, in a foodweb [36]. In a trophic chain the equations have the form
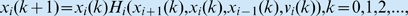
(2)where *ν_i_*(*k*) is a random variable that assumes values for each time step *k* drawn at random from an appropriate distribution *D_i_*(μ*_i_*,σ*_i_* ^2^) with mean and variance parameters μ*_i_* and σ*_i_* ^2^ that can also be estimated during the data fitting process. Adding a stochastic component allows process specification error and environmental stochasticity to be included [38]–[40].

A common, but problematic way to generate *H_i_* as a discrete-time approximation of the continuous-time consumer-resource equations (Box 1), uses the approximation 
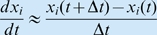
 with *t* = *k* and Δ*t* = 1 combined with additive noise ν, to obtain the relationship (omitting the argument in *k*) between Equations (1) (Box 1) and (2)
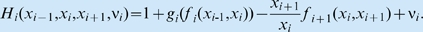
If the time step is too large, this approach may result in *H_i_*<0, which makes no biological sense. To avoid this inconsistency, discrete time models are formulated in terms of the so-called *R*-function [41] defined by *R_i_*(*x_i_*_−1_,*x_i_*,*x_i_*_+1_,ν) = log[*H_i_*(*x_i_*_−1_,*x_i_*,*x_i_*_+1_,ν)]. Thus, if we now take the logarithm on both sides of Equation (2), we obtain the relationship
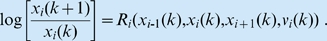

Various forms for *R_i_* have been proposed over the past half-century (as reviewed elsewhere [41],[42]). Until recently standard practice has been to use regression or maximum likelihood techniques [38] to fit the parameters characterizing the functional forms of *R_i_*, which is particularly straight forward if *R_i_* is linear in all its arguments. More comprehensive approaches known generally as hierarchical statistical modelling [43] incorporate dynamic state-space models [44] that are generalizations of Equation (2) and describe the computation of each variable *x_i_*(*k*+1), *i* = 1,…,*n*, in terms of all variables *x_i_*(*k*), *i* = 1,…,*n*. In such formulations the so-called true states {*x_i_*(1), *x_i_*(2),…, *x_i_*(*T*)}, *i* = 1,…,*n*, are not necessarily observable, but rather the data used to fit the parameters in the state space approach consist of sets of observable states{*y_j_*(1), *y_j_*(2),…, *y_j_*(*T*)}, *j* = 1,…,*m*, [39],[43] that are assumed to be related to the true state variables through equations
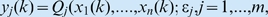
where *ε_j_* are random variables with known distributions (i.e., in terms of an assumed parametric form with estimated mean and variance). This generalization allows the observation errors of the process to be incorporated into the state-space method of parameter estimation [38]–[40],[42],[43] (which in the context of linear systems theory goes back to Kalman filtering developed in the late1950s [45]).

Whatever the particular functional form of the *H_i_*, *i* = 1,…,*n*, and *Q_j_*, *j* = 1,…,*m*, that characterize the problem, the regression approach discussed above is slowly but surely being replaced by Markov Chain Monte Carlo (MCMC) state-space model parameter estimation methods [39],[43], with one study showing this approach to being superior to more traditional approaches [38]. An MCMC methodology referred to as BUGS (Bayesian inference Using Gibbs Sampling) [39],[43] is becoming increasingly accessible to the general scientific community through canned statistical procedures available on open-source software platforms such as R (RBUGS) and also in Windows environments (WinBUGS, as used by Holdo et al. [10]).

## Disease and Shifts of Ecosystem Regimes

Holdo et al. [10] are not the first to recognize the critical role that disease can play in precipitating regime shifts in ecological systems. For example, it was recently shown that an outbreak of canine parvovirus in the Isle Royale wolf population switched the wolf-moose-vegetation trophic chain from top-down to bottom-up control, in the process revealing new levels of bottom-up control that could only be explained by effects arising from climate change [18]. This finding led to the conclusion that predators may play a role in dampening the effects of climate change on the dynamics of their prey. A similar conclusion was reached regarding the role of wolves in dampening the effects of seasonal cycles on scavenger populations [19]. Thus should endemic parvovirus or canine distemper erupt in Yellowstone wolves to the point where it significantly reduces wolf numbers, it could have implications for both the spread of aspen forests and the well-being of coyote, corvid, and eagle scavengers.

The unusual aspect of Holdo et al.'s study [10] is that it analyzes a switch in ecosystem regime arising from the suppression rather than the irruption of a cascading process related to disease (although to begin with humans did cause rinderpest to sweep through eastern and southern Africa in the 1890s). Not all disease-induced changes, however, cascade from upper to lower trophic levels. For example, outbreaks of plague in black-tailed prairie dogs can be traced to increased precipitation stimulating primary production that, in turn, results in increased numbers of rodents and hence fleas and the ensuing transmission of the sylvatic plague-causing bacterium *Yersinia pestis* [20].

## Regime Shift in the Serengeti

**Figure 2:**
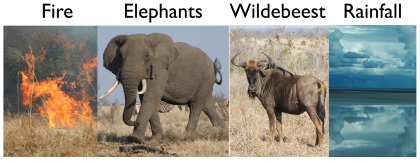
Elephants, fire, wildebeest, and rainfall all have the potential to affect the woody component of African savanna systems. The effect of elephants is through regular browsing and coppicing of trees, fire through episodic burns linked to fuel load, wildebeest after being released from the suppressing effects of endemic rinderpest (a morbillivirus of artiodactyls), and rain through its connections to all system components. Holdo et al. [10] demonstrate that eradication of rinderpest is responsible for the Serengeti switch from a net source to net accumulator of carbon.

Holdo et al. [10] used state-space parameter estimation methods to fit over 44 y of time-series data to two sets of discrete-time trophic interaction models (Box 2). In the first set of models they assessed how both wildebeest (as grazers) and the ratio of wet∶dry season rainfall influences the frequency and intensity of fires. In the second set of models they investigated the influence of fire, elephants, wildebeest, and rain on tree density (Figure 2). Their underlying question is the degree to which the suppression of rinderpest in the early 1960s and the ensuing release of the wildebeest population produced a trophic cascade responsible for switching the Serengeti from a predominantly grassland to a more wooded regime. In particular, they compared the effects of this cascade with those of elephants and fire in mediating the tree-grass balance that characterizes different African savanna systems [14],[21]–[23].

**Figure 3:**
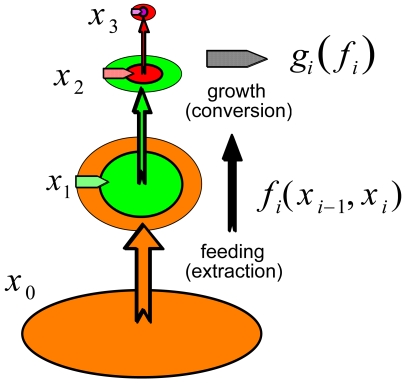
A tritrophic chain in which plants (*x*_1_, green) at the lowest trophic level extract resources (photons, nutrients— *x*_0_, orange) from an underlying flux or pool at a per unit rate *f_i_*(*x_i_* _−1_, *x_i_*), with *i* = 1. The growth rate of the plants is given by *g_i_*(*f_i_*), *i* = 1, while it is consumed by herbivores at the next trophic level at per unit herbivore rate *f_i_*(*x_i_*_−1_, *x_i_*), with *i* = 2. As a result the total rate of change of plant biomass at trophic level 1 is given by the differential equation in Box 1, setting *i* = 1. The same equation applies to the herbivores (*x*_2_, red) at the second trophic level, except now *i* = 2, and to the predators (*x*_3_, black) at the third trophic level, except now *i* = 3 (with *x*_4_ identically 0).

## Conclusion

Rapid land-use change in Africa is creating ever-smaller islands of wild savanna habitat in a sea of engineered landscapes. Coupled with climate change and the threat of emerging diseases [24]–[26], this land-use change makes it increasingly important that we investigate as broadly as possible the possible effects of our wildland management practices for fear of unintended reductions to biological diversity. As long as ecological change occurs more rapidly than evolutionary forces can create new species to replace those going extinct—which is certainly the case with regards to mega-faunal loss during the late Pleistocene era and the more recent loss of vertebrates (particularly amphibians [27])—any major perturbation of existing ecosystems is likely to lead to loss of diversity. Holdo et al. [10] provide an insightful assessment that an unanticipated consequence of vaccinating cattle for rinderpest in east Africa is the current transformation of Serengeti grasslands to woodlands, with ensuing consequence for storage of carbon with implications for climate change and biodiversity. They used statistically powerful parameter estimation methods (Box 2) to identify these effects and, in doing so, illustrated the pitfalls of not analysing the full implications of management actions at a systems level. To avoid such pitfalls in future interventions, we need to pay more attention to developing state-space models (Boxes 1 and 2), with parameters estimated using hierarchical methods (Box 2) to better assess the impacts of intervention on trophic webs as a whole. We also need to extend our models to take account of spatial heterogeneity—an issue not considered in Holdo et al. [10] or in this primer, but to which increasing attention is now being paid [28],[29]. Finally, we need to use our models to assess more deeply than we have in the past how seemingly sensible management actions can have unintended consequences if we are to minimize biodiversity loss from such actions.
